# Identification of Resilient and At-Risk Neighborhoods for Cardiovascular Disease Among Black Residents: the Morehouse-Emory Cardiovascular (MECA) Center for Health Equity Study

**DOI:** 10.5888/pcd16.180505

**Published:** 2019-05-09

**Authors:** Jeong Hwan Kim, Tené T. Lewis, Matthew L. Topel, Mohamed Mubasher, Chaohua Li, Viola Vaccarino, Mahasin S. Mujahid, Mario Sims, Arshed A. Quyyumi, Herman A. Taylor, Peter T. Baltrus

**Affiliations:** 1 Department of Medicine, Division of Cardiology, Emory University School of Medicine, Atlanta, Georgia; 2 Department of Epidemiology, Rollins School of Public Health, Emory University, Atlanta, Georgia; 3 Department of Community Health and Preventive Medicine, Morehouse School of Medicine, Atlanta, Georgia; 4 National Center for Primary Care, Morehouse School of Medicine, Atlanta, Georgia; 5 Division of Epidemiology, School of Public Health, University of California, Berkeley, Berkeley, California; 6 Department of Medicine, University of Mississippi Medical Center, Jackson, Mississippi; 7 Department of Medicine, Morehouse School of Medicine, Atlanta, Georgia

## Abstract

**Introduction:**

Despite the growing interest in place as a determinant of health, areas that promote rather than reduce cardiovascular disease (CVD) in blacks are understudied. We performed an ecologic analysis to identify areas with high levels of CVD resilience and risk among blacks from a large southern, US metropolitan area.

**Methods:**

We obtained census tract–level rates of cardiovascular deaths, emergency department (ED) visits, and hospitalizations for black adults aged 35 to 64 from 2010 through 2014 for the Atlanta, Georgia, metropolitan area. Census tracts with substantially lower rates of cardiovascular events on the basis of neighborhood socioeconomic status were identified as resilient and those with higher rates were identified as at risk. Logistic regression was used to estimate the odds ratios (OR) and 95% confidence intervals (CIs) of being classified as an at-risk versus resilient tract for differences in census-derived measures.

**Results:**

We identified 106 resilient and 121 at-risk census tracts, which differed in the rates per 5,000 person years of cardiovascular outcomes (mortality, 8.13 vs 13.81; ED visits, 32.25 vs 146.3; hospitalizations, 26.69 vs 130.0), despite similarities in their median black income ($46,123 vs $45,306). Tracts with a higher percentage of residents aged 65 or older (odds ratio [OR], 2.29; 95% CI, 1.41–3.85 per 5% increment) and those with incomes less than 200% of the federal poverty level (OR, 1.19; 95% CI, 1.02–1.39 per 5% increment) and greater Gini index (OR, 1.56; 95% CI, 1.19– 2.07 per 0.05 increment) were more likely to be classified as at risk than resilient neighborhoods.

**Discussion:**

Despite matching on median income level, at-risk neighborhoods for CVD among black populations were associated with a higher prevalence of socioeconomic indicators of inequality than resilient neighborhoods.

**Summary**

**What is already known about this topic?**

Residential neighborhood and neighborhood socioeconomic status (SES) are important determinants of cardiovascular disease (CVD) outcomes. It remains understudied what types of neighborhoods promote resilience or increase risk of CVD beyond the effect of neighborhood SES, especially among black Americans, who have a disparately higher prevalence of CVD than white Americans.

**What is added by this report?**

In the Atlanta, Georgia, metropolitan area, using the census tract-level rates of cardiovascular mortality and morbidity for black residents during 2010–2014, we identified 106 resilient neighborhoods and 121 at-risk neighborhoods where black residents had substantially lower-than-expected and higher-than-expected rates of CVD events, respectively, despite similarities in their neighborhood income levels. Yet, certain socioeconomic indicators of inequalities remained important determinants of neighborhood-level CVD risk.

**What are the implications for public health practice?**

Better characterization of resilient and at-risk neighborhood for black Americans helps identify neighborhood-level factors that promote resilience to CVD and helps guide community-level interventions to improve CVD outcomes for black residents in high-risk areas.

## Introduction

Despite the recent, overall reduction in cardiovascular events in the United States, cardiovascular disease (CVD) rates are still higher among black Americans than among white Americans (1,2). Although this interracial disparity in CVD is a public health concern, a substantial degree of intraracial heterogeneity exists within the black population that is often overlooked. More than 50% of black Americans have no form of CVD or cardiovascular risk factors (3). Nevertheless, the factors that promote resilience to CVD among blacks are understudied.

Factors that confer cardiovascular resilience are likely multifactorial, consisting of individual and environmental elements (3). Recent studies have demonstrated residential “place” as a determinant of cardiovascular outcomes (4–7). For example, neighborhood characteristics such as food access, aspects of the built environment, safety, and social cohesion have been individually linked with the cardiovascular health of the residents (7). Furthermore, across racial groups, there is significant variability in CVD by national (6,8) and regional geographic locations (5,9). This geographic variability suggests that certain residential contexts promote cardiovascular health while others increase cardiovascular risk and disease. A better characterization of the spatial contexts that positively promote cardiovascular health (ie, areas with cardiovascular resilience, particularly for black residents), is important in understanding the CVD burden for black Americans and guiding interventions to improve outcomes among them.

We investigated the resilience of neighborhoods against expected CVD rates among black adults in Atlanta, Georgia. By using census tract–level cardiovascular mortality and morbidity rates, we identified neighborhoods that were resilient or at risk for CVD among black residents. Specifically, we identified resilient and at-risk neighborhoods that were not predominantly confounded by differences in neighborhood socioeconomic status (SES), an established determinant of cardiovascular outcomes (7,10–12). Lastly, we conducted an ecologic-level analysis of the census-derived measures to identify the characteristics that distinguish resilient and at-risk areas.

## Methods

**Figure 1:**
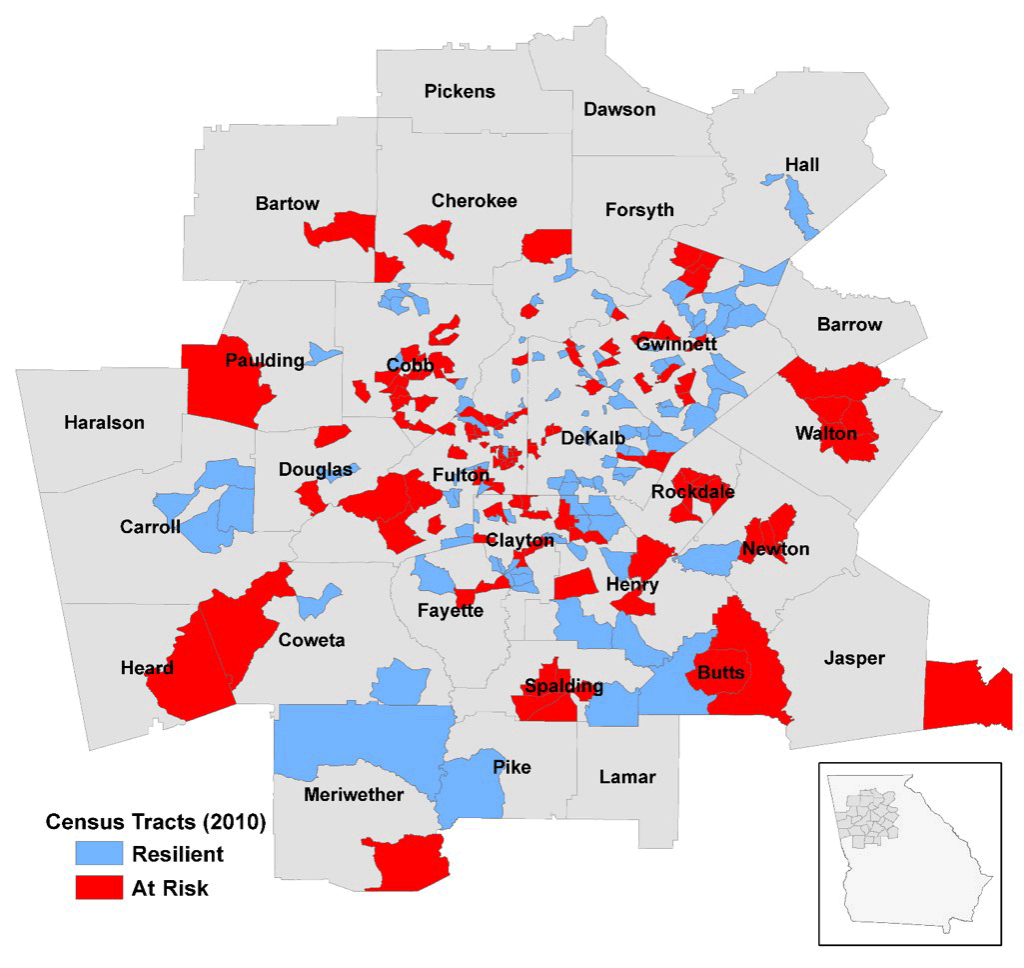
Study region of the Morehouse–Emory Cardiovascular Center for Health Equity project conducted in the Atlanta, Georgia, metropolitan area with 2010 census tract boundaries. Resilient and at-risk census tracts identified by the residual percentile method are indicated.

**Geographic region of the study**. This study was completed as part of the Morehouse–Emory Cardiovascular (MECA) Center for Health Equity project. Census tract was used as the unit of analysis. Data were obtained and analyzed for the 992 census tracts in the 36-county Atlanta–Athens–Clarke–Sandy Springs combined statistical area that makes up the Atlanta metropolitan area (Figure 1).

**Mortality data.** Cardiovascular mortality data for the 5-year period from 2010 through 2014 were obtained from the Georgia Department of Public Health. We received the counts of all deaths attributable to cardiovascular causes (identified as ICD 10 codes I00–I78, from the *International Classification of Diseases, Tenth Revision* [13] or ICD 9 codes 390-434 and 436–448 from the *International Classification of Diseases, Ninth Revision* [14]) for blacks aged 35 to 64, the age group that captured most of the population with CVD risk while excluding those aged 65 or older to minimize the confounding by noncardiac comorbidities. Counts for census tracts with fewer than 5 deaths were censored for confidentiality reasons, which resulted in a total of 347 census tracts with uncensored data. Additionally, to minimize the number of census tracts censored because of few events and to ensure stable events rates over the 5-year period, only the tracts with at least 200 black adults aged 35 to 64 were included (N = 346). Counts of deaths were then divided by the black population aged 35 to 64 living in the respective census tracts (2010 US Census data) (15) to generate the mortality rate for each census tract. The rates were reported as the number of events per 5,000 person-year (per 1,000 people over the 5-year period).

**Morbidity data.** Cardiovascular morbidity data from 2010 through 2014 were obtained from the Georgia Hospital Association. We obtained aggregated counts of emergency department (ED) visits and hospitalizations for cardiovascular reasons, identified with ICD 10 codes I00–I78 (13) or ICD 9 codes 390–434 and 436–448 (14) for blacks aged 35 to 64 from 2010 through 2014. Census tracts with fewer than 6 events were censored for confidentiality reasons, resulting in 802 tracts with uncensored data for ED visit and 763 tracts for hospitalization data. As with mortality, only tracts with at least 200 black adults aged 35 to 64 were included (N = 693 for ED visits; N = 675 for hospitalizations). Counts of ED visits and hospitalizations were divided by the population of blacks aged 35 to 64 living in the respective census tract (2010 US Census data) (15) to calculate the rates of hospitalization and ED visits for each census tract. The rates were reported as the number of events per 5,000 person-year.

**Census-derived measures.** We obtained census tract data from the 2010 US Decennial Census (15) to characterize the demographic and socioeconomic composition of the identified at-risk and resilient census tracts. The variables selected included factors that have been previously linked with CVD, such as SES and housing-related indicators (5,10,16), and measures of demographic composition. Demographic data obtained were percentage female, black median age, percentage aged 65 or older, percentage aged 17 or younger, percentage minority population, percentage black population, percentage speaking English less than well, percentage of single-parent households, and percentage civilians with a disability. For the measures of SES, we obtained median black household income, percentage education certifications (high school, college), percentage unemployed, percentage with incomes below the federal poverty level, percentage with incomes below 200% of the federal poverty level (ie, percentage of the population with income below twice the federal poverty level, as an index of the proportion in or near poverty), and Gini index (17) (a measure of income inequality from perfect equality [0], where everyone receives the same income, to perfect inequality [1], where a single person receives the total income of the community). For housing-related measures, median home value, percentage living in multi-unit structures, percentage living in mobile homes, percentage living in crowded units (defined as housing units occupied by more than 1 person per room), and percentage living in group quarters. Finally, the percentage of households without a vehicle was assessed as a measure of transportation accessibility.

**Identification of resilient and at-risk census tracts.** We identified census tracts that were resilient and at risk based on the aforementioned measures of cardiovascular outcomes: deaths, ED visits, and hospitalizations. First, we identified low-rate and high-rate census tracts solely on the basis of the distribution of the outcome measures. A census tract was considered low-rate on one of the 3 measures if its rate was in the bottom quartile of the measure and high-rate if its rate was in the highest quartile of the measure. Then, if a census tract was considered low-rate on at least 2 of the 3 measures and not high-rate for any measure, the tract was classified as a low-rate census tract. Similarly, being labeled as a high-rate tract on at least 2 of the 3 measures and not low-rate on any measure classified the tract as high-rate.

**Figure 2:**
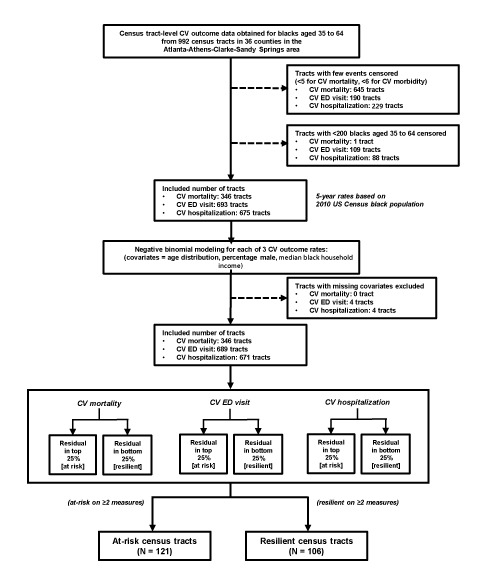
The steps in the identification of at-risk and resilient census tracts by the residual percentile method. Census tract-level CV outcome data for blacks aged 35 to 64 from 992 census tracts in 36 counties in the Atlanta–Athens-Clarke–Sandy Springs combined statistical area were used to identify 121 at-risk and 106 resilient census tracts. Abbreviations: CV, cardiovascular; ED, emergency department.

Because it is well documented that neighborhood SES is a strong determinant of cardiovascular outcomes (5,10,11), we identified areas that were not predominantly confounded by differences in neighborhood SES. We used the residual percentile method, which is similar to a method used to by Fry-Johnson et al (18) to identify counties with low infant mortality rates independent of county-level SES. By using this method (Figure 2), we identified census tracts that had substantially lower or higher rates of CVD outcomes than the rates that would be expected on the basis of their neighborhood SES. Census tracts with lower than expected CVD outcome rates were defined as resilient, and those with higher than expected CVD rates were defined as at-risk. To do so, a negative binomial model was built for each of the 3 measures. Each model was adjusted for census tract-level socioeconomic variables for blacks, including age distribution (in 5-year age groups), percentage male, and median black household income. Census tracts without any missing covariate were included in the model (N = 346 for mortality; N = 689 for ED visits; N = 671 for hospitalizations). Census tracts with model residuals in the highest 25% (substantially higher rates than predicted) were considered at risk for the measure. Similarly, tracts with model residuals in the lowest 25% (substantially lower rates than predicted) were considered resilient for the measure. Census tracts at risk or resilient on at least 2 of 3 measures were finally labeled as at-risk or resilient census tracts, respectively, and included in our analysis. Any census tract designated at risk for one measure but resilient for any other measures, or vice versa, was excluded.

**Statistical analysis.** We used *t* tests to compare demographic and socioeconomic measures of at-risk and resilient census tracts, which we identified by the residual percentile method. The measures that were significantly different were subsequently analyzed by using logistic regression models. The OR and 95% CI for being labeled at-risk census tracts compared with resilient tracts were estimated in bivariate and multivariable models, for 5% increment in the included census tract measures. We verified absence of any major collinearity among the explanatory variables by computing the condition index (19) in the fully adjusted model (27.49). *P* < .05 was considered significant. Statistical analyses were performed by using SAS version 9.3 (SAS Institute Inc).

## Results

In our initial analyses, unadjusted for neighborhood SES, we identified 130 low-rate and 137 high-rate census tracts. Tracts selected using this approach differed in their CVD outcome measures as expected (mortality: 6.27 for low-rate tracts vs 15.75 for high-rate tracts; ED visits: 27.67 for low-rate tracts vs 159.70 for high-rate tracts; hospitalizations: 21.60 for low-rate tracts vs 165.10 for high-rate tracts; per 5,000 person-year), but they also had substantial difference in the median black household income levels ($60,980 for low-rate tracts vs $29,015 for high-rate tracts). By using the residual percentile method, we identified 106 resilient and 121 at-risk census tracts. The resilient census tracts had lower rates of cardiovascular mortality, hospitalization, and ED visits than the at-risk census tracts, but the median black household income levels of the resilient and the at-risk census tracts did not differ from each other substantially (Table 1). Furthermore, resilient and at-risk census tracts were located throughout the metropolitan Atlanta area without clustering of either resilient or at-risk tracts, and resilient and at-risk census tracts were also often adjacent to one another (Figure 1).

**Table 1:** Mean Rates of Cardiovascular Outcomes and Median Household Income for Black Residents in Resilient and At-Risk Census Tracts^a^, Atlanta, Georgia, 2010–2014

Variable	Resilient Tract (n = 106)	At Risk Tract(n = 121)	*P* Value
Mortality rate^b^	8.1	13.8	<.001
Emergency department visits^b^	32.3	146.3	<.001
Hospitalization rate^b^	26.7	130.0	<.001
Median household income, $	46,123	45,306	.79

a Selected by the residual percentile method.

b Number of events per 5,000 person-year.

The median age of black residents was similar in resilient and at-risk census tracts, but the proportion of residents aged 65 or older was significantly lower in resilient census tracts than in at-risk census tracts (*P* < .001) (Table 2). The proportion of women and black residents were also similar in both neighborhood types. However, fewer civilians with a disability resided in resilient census tracts than in at-risk tracts (*P* < .001).

**Table 2:** Comparison of Demographic, Socioeconomic, Housing and Transportation Characteristics of Resilient and At-Risk Census Tracts, Atlanta, Georgia^a^

Variable	Resilient Tract (n = 106)	At-Risk Tract (n = 121)	*P* Value
**Demographic characteristic**			
% Female	54.8	55.6	.29
Median black age, y	32.3	32.1	.77
% Aged ≥65 y	7.8	10.4	<.001
% Aged ≤17 y	26.4	25.3	.19
% Racial/ethnic minority population	67.7	62.5	.14
% Black population	48.8	45.3	.38
% Speaking English less than well	4.8	4.0	.34
% Single-parent households	13.9	14.0	.88
% Civilians with a disability	9.7	12.0	<.001
**Socioeconomic status of residents**			
Median black income, $	46,123	45,306	.79
% With no high school diploma	13.3	16.3	.02
% With high school diploma or less	34.8	43.3	<.001
% With some college	35.8	32.4	.007
% College graduate	29.4	24.4	.01
% Unemployed	13.2	13.4	.85
% With income below federal poverty level	20.2	22.8	.14
% With income below 200% of federal poverty level	33.7	40.7	.003
Gini index^b^	0.38	0.42	<.001
**Housing**			
Median home value, $	181,761.00	176,008.00	.62
% Multi-unit structure	18.3	13.8	.10
% Mobile home	2.5	2.5	.97
% Crowded unit	3.2	3.1	.96
% Living in group quarter	0.9	1.7	.27
**Transportation: % with no vehicle in household**	7.6	10.8	.02

a Values are mean values of percentage values unless noted otherwise.

b A measure of income inequality from perfect equality (0), where everyone receives the same income, to perfect inequality (1), where a single person receives the total income of the community.

For socioeconomic measures, resilient census tracts had a higher percentage of college graduates and those with some college education than at-risk census tracts (*P* = .01 and .007, respectively). Similarly, there were more people with high school diploma or less in at-risk census tracts than in resilient tracts (*P* < .001). Though the median black household income was similar and the percentage of people with incomes below the federal poverty level were similar in the 2 groups, resilient census tracts had fewer residents with incomes below 200% of the federal poverty level than at-risk census tracts and had significantly lower Gini index than at-risk census tracts (0.38 vs 0.42, *P* < .001). Other housing measures did not differ significantly between resilient and at-risk tracts, but more households in at-risk census tracts had no vehicle than in resilient tracts (*P* = .02).

Six measures that differed significantly (*P* < .05) between resilient and at-risk census tracts were included in regression analyses: percentage aged 65 or older, percentage of civilians with a disability, percentage with no high school diploma, percentage with incomes below 200% of the federal poverty level, Gini index, and percentage with no vehicle in household (Table 3). After simultaneous adjustment in the model, census tracts with a 5% increment in the proportion aged 65 or older were 2.29 times (95% CI, 1.41–3.85) more likely to be categorized as at-risk tracts. Similarly, tracts with 5% increment in the percentage below 200% poverty were 1.19 times (95% CI, 1.02–1.39) more likely to be designated as at-risk tracts. Finally, tracts with a 0.05 higher Gini index were 1.56 times (95% CI, 1.19–2.07) more likely to be classified as at-risk tracts.

**Table 3:** Predictors of Census Tracts Being At Risk Versus Resilient (N = 227), Atlanta Metropolitan Area^a^

Variable	Crude	Adjusted
	Odds Ratio (95% Confidence Interval)	
% Aged ≥65 y	2.11 (1.51–3.03)^b^	2.29 (1.41–3.85)^b^
% With disability	1.77 (1.31–2.43)^b^	1.12 (0.70–1.81)
% With no high school diploma	1.19 (1.03–1.38)^b^	0.98 (0.79–1.22)
% With annual income below 200% of federal poverty level	1.12 (1.04–1.22)^b^	1.19 (1.02–1.39)^b^
Gini index^c^, per 0.05 increment	1.59 (1.28–2.02)^b^	1.56 (1.19 -2.07)^b^
% With no vehicle in household	1.17 (1.02–1.35)^b^	0.82 (0.66–1.02)

a Crude and adjusted odds ratios of being classified as an at-risk census tract versus a resilient census tracts are shown for 5% increments in each of the examined factors except for Gini index (per 0.05 unit increment).

b Significant (*P* < .05) results.

c A measure of income inequality from perfect equality [0], where everyone receives the same income, to perfect inequality [1], where a single person receives the total income of the community.

## Discussion

We identified several demographic and socioeconomic indicators of income and education inequality at the ecologic level that distinguished at-risk neighborhoods from resilient neighborhoods; having a higher proportion of residents aged 65 or older and residents with income below 200% of the federal poverty level and greater income inequality were independent factors that separated at-risk neighborhoods from resilient neighborhoods. To our knowledge, this study is the first to use census tract–level data to identify areas resilient to and at risk for CVD for black residents in a large US metropolitan area.

Our approach to identify resilient and at-risk neighborhoods was unique in that we quantified the deviation of cardiovascular mortality and morbidity for neighborhoods from what would be predicted on the basis of their neighborhood SES. Over the past 2 decades, studies have demonstrated that living in socioeconomically disadvantaged neighborhoods is associated with a greater burden of cardiovascular risk and disease (7,12). This association has been demonstrated not only with cardiovascular risk factors (11,20,21), but also with incidence of CVD (5,22) and cardiovascular mortality (10,23). However, despite the growing interest in neighborhoods as a determinant of health, less is known about outlier communities that have an unusually lower or higher burden of CVD than what would be expected given their socioeconomic composition. Understanding of those outlier communities will elucidate neighborhoods’ health-promoting factors better than using SES.

Reports of such outlier communities date back as early as the 1960s (24), but contemporary data from the United States is still largely lacking. The bulk of available evidence on resilient neighborhood comes from research in Europe (25–28) and New Zealand (29), in which neighborhoods with higher or lower rates of all-cause mortality and morbidity than predicted from neighborhood SES were identified, similar to the approach we used in this analysis. However, our analysis differed from these reports in 2 major aspects. First, we examined cardiovascular-specific mortality and morbidity whereas the other studies examined all-cause mortality or morbidity. As previously reported (27), the resilience of neighborhoods may differ depending on the etiologies of mortality, and examination of cause-specific mortality and morbidity as in our analysis helps identify potential mechanistic pathways between neighborhood characteristics and CVD more directly. Second, previous studies extracted mortality and morbidity data from the entire population of the examined communities, potentially masking the racial/ethnic differences in the association between neighborhoods and individuals. On the other hand, we focused on a specific racial group, blacks, to explore the intraracial differences between types of neighborhood on CVD and eventually to help design effective interventions to improve neighborhoods for better cardiovascular outcomes of among black residents.

We also identified several independent features that distinguished resilient and at-risk neighborhoods for CVD in black residents. Not only do these factors illustrate the primary ecologic-level determinants of neighborhood resilience or risk for CVD for black residents, but they also could provide insights into policy design or community-level interventions to improve cardiovascular outcomes among blacks. First, despite similarities in the median age and the proportion of population aged 17 or younger, at-risk census tracts had a higher proportion of residents aged 65 or older than resilient census tracts. A similar finding was also previously reported in relation to all-cause mortality (26). Interestingly, the cardiovascular outcome data used in our analysis did not include people aged 65 or older. Thus, although an older age is a known risk factor for cardiovascular mortality and morbidity (30), the proportion of those aged 65 or older likely represents a proxy for contextual factors of the at-risk neighborhood environment. For example, a higher proportion of elderly residents may correlate with a stagnant or declining overall population with fewer middle-aged working residents, whereas a greater influx of residents, likely with more economic opportunities, may be associated with resilient neighborhoods (29,31). Further characterization of the population composition with trajectory may help further elucidate the significance of the percentage of the elderly in the CVD resilience and risk of the overall neighborhood.

Secondly, both a higher proportion of those with incomes under 200% of the federal poverty level and greater income inequality were also independently associated with at-risk neighborhoods compared with resilient neighborhoods. Although the median black income and percentage of those under the poverty level were similar in resilient and at-risk neighborhoods, our results suggest that even moderate deprivation of income (ie, those in the near-poverty and the resultant income equality despite similarities in the median income) could adversely affect CVD outcomes among black residents. In addition to the level of neighborhood income itself (7,12), income inequality has been previously associated with CVD burden (32,33). Thus, our findings reconfirm that socioeconomic deprivation, even at a moderate degree, may affect cardiovascular resilience and risk at the ecologic level. Whether income deprivation and inequality represent proxies for other contextual factors of neighborhoods remains to be investigated. Although limited in our analysis, further characterization of people with incomes at the poverty or near-poverty level would be important, because they may be the vulnerable population that would most benefit from the appropriate aid to improve their cardiovascular outcomes or prevention measures.

Our study has limitations. Because of its cross-sectional design, any inference of causation from the observed findings is limited. Longitudinal analyses of the neighborhood resilience and the neighborhood-level cardiovascular outcomes would be needed. Furthermore, the definition of neighborhood in a fixed unit of census tracts may have masked variability of smaller communities and residential contexts. Similar analysis in smaller units, such as census block, may be informative to validate or augment our analysis. Third, because the data examined were limited at the ecologic level, the subjective, contextual factors of living in a given neighborhood are not accounted for in our analysis. However, our work was undertaken as the first cornerstone of the larger MECA project, which aims for a multilevel exploration of cardiovascular resilience of US black adults and lays a foundation for continued investigation. In the subsequent stages of the MECA project, we plan to examine the characteristics of the identified at-risk and resilient neighborhoods at the individual level, which would enable us to better understand the contextual versus compositional factors contributing risk or resilience to the residents of the selected tracts.

In conclusion, by using neighborhood-level data on cardiovascular mortality and morbidity for black residents, we identified resilient and at-risk neighborhoods for CVD among black adults in a large southern US city. These resilient and at-risk neighborhoods substantially differed in the rates of cardiovascular mortality and morbidity despite their similar income levels, suggesting that they represent a distinct residential context, or place, that promotes or jeopardizes the cardiovascular health of its black residents beyond the effect of neighborhood SES. However, even with our definitions of resilient and at-risk neighborhoods, certain socioeconomic indicators of inequality remained important predictors of CVD risk at the neighborhood level. Further exploration of contextual factors other than neighborhood SES are needed to fully characterize the factors that constitute a residential place that either promotes or threatens the cardiovascular health of its black residents.
